# Determination of geometric information and radiation field overlaps on the skin in percutaneous coronary interventions with computer‐aided design‐based X‐ray beam modeling

**DOI:** 10.1002/acm2.13457

**Published:** 2021-10-26

**Authors:** Atsushi Fukuda, Pei‐Jan P. Lin, Nao Ichikawa, Kosuke Matsubara

**Affiliations:** ^1^ Department of Radiological Sciences, School of Health Sciences Fukushima Medical University Fukushima Japan; ^2^ Department of Radiology Virginia Commonwealth University Medical Center Richmond Virginia USA; ^3^ Department of Radiological Technology, Faculty of Health Science Kobe Tokiwa University Hyogo Japan; ^4^ Department of Quantum Medical Technology, Faculty of Health Sciences, Institute of Medical, Pharmaceutical and Health Sciences Kanazawa University Ishikawa Japan

**Keywords:** computer‐aided design, percutaneous coronary intervention, X‐ray beam field skin overlap, X‐ray beam modeling

## Abstract

**Purpose:**

This study aimed to develop a method for the determination of the source‐to‐surface distance (SSD), the X‐ray beam area in a plane perpendicular to the beam axis at the entrance skin surface (*A_p_*), and the X‐ray beam area on the actual skin surface (*A_s_*) during percutaneous coronary intervention (PCI).

**Materials and Methods:**

Male and female anthropomorphic phantoms were scanned on a computed tomography scanner, and the data were transferred to a commercially available computer‐aided design (CAD) software. A cardiovascular angiography system with a 200 × 200 mm flat‐panel detector with a field‐of‐view of 175 × 175 mm was modeled with the CAD software. Both phantoms were independently placed on 40 mm thick pads, and the examination tabletop at the patient entrance reference point. Upon panning, the heart center was aligned to the central beam axis. The SSD, *A_p_*, and *A_s_* were determined with the measurement tool and Boolean intersection operations at 10 gantry angulations.

**Results:**

The means and standard deviations of the SSD, *A_p_*, and *A_s_* for the male and female phantoms were 573 ± 15 and 580 ± 15 mm, 8799 ± 1009 and 9661 ± 1152 mm^2^, 10495 ± 602 and 11913 ± 600 mm^2^, respectively. The number of *A_s_* overlaps for the male and female phantoms were 15/45 and 21/45 view combinations, respectively.

**Conclusions:**

CAD‐based X‐ray beam modeling is useful for the determination of the SSD, *A_p_*, and *A_s_*. Furthermore, the knowledge of the *A_s_* distribution helps to reduce the *A_s_* overlap in PCI.

## 1 INTRODUCTION

Percutaneous coronary intervention (PCI) is a promising technique that is extensively performed for the treatment of coronary artery disease.^1^, ^2^ However, the radiation doses to the patients increase according to the complexity of the coronary disease such as the previous history of coronary artery bypass grafting, body mass index, the number of treated lesions, and chronic total occlusions.^3^, ^4^ The deterministic effects of the skin have been reported in the forms of erythematous reaction, ulceration, and necrosis,^5^, ^6^ and the radiation doses to skin and the locations should be monitored to avoid radiation‐induced skin injuries in PCI.^6^, ^7^, ^8^, ^9^

The angiography system displays incident air‐kerma value ($K_{a,i}$) and the rate value (${\dot{K}}_{a,i}$) at patient entrance reference point (PERP) and the kerma area product value ($P_{\mathrm{KA}}$) according to the IEC 60601‐2‐43 in 2000, and the amendments of 2010 and 2019.^10^ The PERP is defined at a distance of 15 cm from the isocenter to the X‐ray tube along the central beam axis.^10^ Thus, the PERP rotates along with the C‐arm rotation during PCI, and does not accurately correspond to the patient entrance skin surface.^11^, ^12^ Two methods are used to obtain entrance surface air kerma ($K_{a,e}$) values.

One of them is the distance correction based on the source–surface distance (SSD) expressed according to the following equation:

(1) $$K_{a,e}=K_{a,i}\;Bk_{ta}\frac{SPD^{2}}{SSD^{2}}.$$

$K_{a,e}$ can be calculated from $K_{a,i}$ using the appropriate backscatter factor *B*, examination table (and pad) attenuation correction factor $k_{\mathrm{ta}}$, and source‐to‐PERP distance (SPD). However, because the examination table and the C‐arm angulations are moved according to the heart position and/or the target lesion, the determination of the SSD is not an easy task in clinical setting. The other is the beam field correction conducted based on the area of the X‐ray beam in a plane perpendicular to the beam axis at the entrance of the skin surface (*A_p_*) according to the following equation:

(2) $$K_{a,e}=\frac{P_{KA}}{A_{p}}\;Bk_{ta.}$$

However, because the *A_p_* varies as functions of the SSD, source‐to‐image receptor distance (SID), the selected field‐of‐view (FOV), and the beam collimation, the determination of *A_p_* is not straightforward in the clinical setting.

Numerous radiation dose mapping softwares have been recently released commercially, and have been applied for the radiation dose assessment in PCI.^12^, ^13^ However, these softwares do not focus on the geometrical parameters for the determination of the value of the X‐ray beam field on the actual skin surface (*A*_s_). Recent improvements of a medical imaging computer workstation in radiology allow the conversion of the data from digital imaging and communications in medicine (DICOM) format to the stereolithography (STL) that has been commonly used in additive manufacturing.^14^ The STL data consist of multiple triangular meshes, and provide increased geometrical accuracy and the visualization in computer‐aided design (CAD) software. We hypothesized that it would be able to determine the SSD, *A_p_*, and *A_s_* in the CAD software in which the angiography system was accurately modeled. The SSD or *A_p_* can be used for the correction of the $K_{a,e}$ from $K_{a,i}$ or $P_{\mathrm{KA}}$. Furthermore, the *A_s_* helps to determine the overlaps of the radiation fields on the actual skin surface. To the best of our knowledge, there have been no published methods for the calculation of the SSD, *A_p_*, and *A_s_* with the CAD software in conjunction with patient‐specific computed tomography (CT) data. The aim of this study was the development of the method for the determination of these geometrical values in PCI.

## 2 MATERIALS AND METHODS

### 2.1 Patient phantom modeling

**FIGURE 1:**
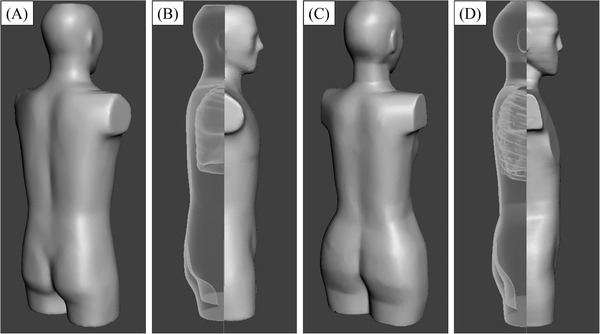
STL data settings for male and female adult anthropomorphic phantoms. The male and female phantoms were scanned in a CT system, and the DICOM data were converted to STL data (A: male, C: female) in the workstation. The hollow tool was used to create internal cavities inside the STL data with a consistent wall (skin) thickness of 1 mm (B: male, D: female). The dorsal sides of the male (B) and female STL data (D) are depicted as transparent for illustration purposes. CT, computed tomography; DICOM, digital imaging and communications in medicine; STL, Stereolithography

A SOMATOM Definition Flash CT scanner (Siemens Healthcare, Forchheim, Germany) was employed to scan an anthropomorphic adult male (ATOM Adult Male Model 701, CIRS Inc., Norfolk, VA, USA) and an adult female phantom (ATOM Adult Female Model 702; CIRS Inc.) without the dedicated bilateral breast attachments.^15^, ^16^ Heights and weights for the male and female phantoms were 173 and 160 cm, 73 and 55 kg, respectively. The scan parameters included a tube potential of 120 kVp, tube current of 140 mA, total collimation width of 38.4 mm, spiral pitch factor of 0.6, and the data collection diameter of 500 mm. The data for two phantoms were reconstructed with a slice thickness/interval of 1 mm and a soft tissue kernel (B41f), and were transferred to an open‐source workstation, 3D slicer version 4.10.2.^17^ The workstation was employed to convert the data from the DICOM to the STL formats. Furthermore, the STL data were transferred to dedicated three‐dimensional modeling software (Meshmixer, Autodesk, San Rafael, CA, USA). The hollow tool was used to create internal cavities inside the STL data with a consistent wall (skin) thickness of 1 mm (Figure 1).

**FIGURE 2:**
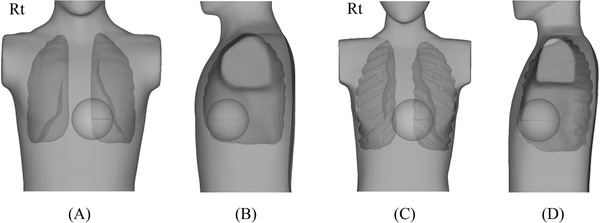
Heart locations for male and female anthropomorphic phantoms. The heart was modeled as a sphere with a diameter of 10 cm. The spherical centers for male/female phantoms were located at the left lateral distances from the midline (*x*‐axis) of 30 mm/20 mm, at 77 mm/66 mm (1/3 depth) from the frontal skin surface (*y*‐axis), and at the center from the left atrial appendage to the diaphragm (*z*‐axis). Anterior views for male (A) and female (C) phantoms. Lateral views for male (B) and female (D) phantoms

The male and female STL data were imported in free‐ and open‐source CAD software, FreeCAD version 0.17.^18^ The heart was modeled as a sphere with a diameter of 10 cm, and the centers for the male/female phantoms were located at the left lateral distances of 30/20 mm from the midline (*x*‐axis), at 77/66 mm (1/3 depth) from the frontal skin surfaces (*y*‐axis), and at the centers from the left atrial appendage to the diaphragm (*z*‐axis) (Figure 2).^15^, ^16^, ^19^, ^20^

### 2.2 Modeling of the cardiovascular angiography system

The angiography equipment was modeled to emulate the commercially available cardiovascular angiography system that comprises a 200 × 200 mm flat‐panel detector (FPD) (Infinix Celeve‐i, Canon Medical Systems, Nasu, Japan). The geometrical parameters employed were as follows: source‐to‐isocenter distance, 700 mm; X‐ray focus size, 1.2 mm diameter circle; image receptor cover‐to‐FPD distance, 50 mm; FOV, 175 × 175 mm; examination tabletop, PERP; and a pad lying on the examination table, 40 mm.

### 2.3 Determinations of the panning displacement, SID, SSD, *A_p_*, and *A_s_*

The coordinate origin for *x*‐, *y*‐, and *z*‐axes was set at the isocenter of the C‐arm. Ten typical clinical projections were employed in this study (Tables 1, 2, 3, 4). All of the phantoms were placed (independently) on the examination table, and were moved (panned) so that the heart centers were shifted in the central beam axis at these working angles. The lateral displacements (LD) and cranio‐caudal displacements (CCD) were recorded as the panning displacements. Positive LD and CCD values indicate left and cranial movements, respectively. Furthermore, the total panning was calculated as $\sqrt{LD^{2}+CCD^{2}}$. The SID was determined so that the image receptor cover and the phantom surface were separated by 100 mm. The SSD was also calculated as the distance from the X‐ray focus to the actual skin surface along the central beam axis. The *A_p_* and *A_s_* values were determined based on the calculation of the beam dimensions at the entrance skin surface and the cross‐section dimensions between the beam and the actual skin surface using the Boolean intersection operation, respectively. Furthermore, the overlaps of the *A_s_* were calculated with these *A_s_* values measured in all X‐ray angulations. A total of 10 typical angulations in PCI study were simulated. Therefore, 10 *A_s_* were superimposed on the skin surface of the male and female phantoms.

**TABLE 1:** SSD, *A_p_*, and *A_s_* for male anthropomorphic phantom as a function of X‐ray tube angulation

X‐ray tube angulation (°)	Table height* (cm)	Panning LD** (mm)	Panning CCD*** (mm)	Total panning (mm) $\sqrt{LD^{2}+CCD^{2}}$	SID (mm)	SSD (mm)	*A_p_* (mm^2^)	*A_s_* (mm^2^)	*A_s_*/*A_p_*
PA 0°	15	0	0	0	976	593	10 738	10 991	1.02
RAO 10°/CAU 30°	15	−8	−28	29	1049	571	8639	10 430	1.21
RAO 30°/CAU 30°	15	−28	−32	43	1083	570	8083	9759	1.21
RAO 30°	15	−28	0	28	1024	590	9668	10 800	1.12
RAO 30°/CRA 30°	15	−28	32	43	1066	581	8661	11 016	1.27
LAO 10°/CRA 30°	15	8	28	29	1016	587	9720	11 006	1.13
LAO 30°/CRA 30°	15	28	32	43	1052	567	8472	11 177	1.32
LAO 45°/CRA 30°	15	48	39	62	1105	558	7448	9574	1.29
LAO 45°	15	48	0	48	1028	568	8898	10 425	1.17
LAO 45°/CAU 30°	15	48	−39	62	1068	547	7659	9768	1.28
Mean ± SD	NA	NA	NA	39 ± 18	1047 ± 37	573 ± 15	8799 ± 1009	10 495 ± 602	1.20 ± 0.09

*A_p_*, area of the X‐ray beam in a plane perpendicular to the beam axis at entrance skin surface; *A_s_*, area of the X‐ray beam field on the actual skin surface; CAU, caudal; CCD, cranio‐caudal displacement; CRA, cranial; LAO, left anterior oblique; LD, lateral displacement; PA, posterior‐anterior; RAO, right anterior oblique; SD, standard deviation; SID, source–image receptor distance; SSD, source–surface distance.

* Table height indicates the distance from the isocenter to the examination tabletop.

** Positive LD values indicate the left lateral displacement.

*** Positive CCD values indicate the cranial displacement.

**TABLE 2:** SSD, *A_p_*, and *A_s_* values for female anthropomorphic phantom as functions of X‐ray tube angulation

X‐ray tube angulation (°)	Table height* (cm)	Panning LD** (mm)	Panning CCD*** (mm)	Total panning (mm) $\sqrt{LD^{2}+CCD^{2}}$	SID (mm)	SSD (mm)	*A_p_* (mm^2^)	*A_s_* (mm^2^)	*A_s_*/*A_p_*
PA 0°	15	0	0	0	947	599	11 629	11 914	1.02
RAO 10°/CAU 30°	15	−5	−16	17	1027	571	9009	11 405	1.27
RAO 30°/CAU 30°	15	−16	−18	24	1055	570	8513	11 556	1.36
RAO 30°	15	−16	0	16	982	596	10 716	12 843	1.20
RAO 30°/CRA 30°	15	−16	18	24	1020	589	9710	12 838	1.32
LAO 10°/CRA 30°	15	5	16	17	973	595	10 876	12 030	1.11
LAO 30°/CRA 30°	15	16	18	24	1009	576	9492	12 161	1.28
LAO 45°/CRA 30°	15	27	22	35	1054	570	8528	11 350	1.33
LAO 45°	15	27	0	27	988	579	9997	11 985	1.20
LAO 45°/CAU 30°	15	27	−22	35	1043	551	8143	11 055	1.36
Mean ± SD	NA	NA	NA	22 ± 10	1010 ± 37	580 ± 15	9661 ± 1152	11 913 ± 600	1.24 ± 0.11

*A_p_*, area of the X‐ray beam in a plane perpendicular to the beam axis at entrance skin surface; *A_s_*, area of the X‐ray beam field on the actual skin surface; CAU, caudal; CCD, cranio‐caudal displacement; CRA, cranial; LAO, left anterior oblique; LD, lateral displacement; PA, posterior‐anterior; RAO, right anterior oblique; SD, standard deviation; SID, source–image receptor distance; SSD, source–surface distance.

* Table height indicates the distance from the isocenter to the examination tabletop.

** Positive LD values indicate the left lateral displacement.

*** Positive CCD values indicate the cranial displacement.

**TABLE 3:** Overlaps of *A_s_* for male anthropomorphic phantom as a function of the X‐ray tube angulation

	Overlap of *A_s_* (mm^2^)									
X‐ray tube angulation (°)	PA 0°	RAO 10°/CAU 30°	RAO 30°/CAU 30°	RAO 30°	RAO 30°/CRA 30°	LAO 10°/CRA 30°	LAO 30°/CRA 30°	LAO 45°/CRA 30°	LAO 45°	LAO 45°/CAU 30°
PA 0°	NA	–	–	–	–	–	–	–	–	–
RAO 10°/CAU 30°	1455	NA	–	–	–	–	–	–	–	–
RAO 30°/CAU 30°	261	3755	NA	–	–	–	–	–	–	–
RAO 30°	1894	527	948	NA	–	–	–	–	–	–
RAO 30°/CRA 30°	333	0	0	1650	NA	–	–	–	–	–
LAO 10°/CRA 30°	1913	0	0	0	0	NA	–	–	–	–
LAO 30°/CRA 30°	694	0	0	0	0	5182	NA	–	–	–
LAO 45°/CRA 30°	0	0	0	0	0	0	4819	NA	–	–
LAO 45°	0	0	0	0	0	0	49	55	NA	–
LAO 45°/CAU 30°	0	0	0	0	0	0	0	0	56	NA

CAU, caudal; CRA, cranial; LAO, left anterior oblique; PA, posterior‐anterior; RAO, right anterior oblique; *A_s_*, area of the X‐ray beam field on the actual skin surface.

**TABLE 4:** Overlaps of *A_s_* for female anthropomorphic phantom as a functions of the X‐ray tube angulation

	Overlap of *A_s_* (mm^2^)									
X‐ray tube angulation (°)	PA 0°	RAO 10°/CAU 30°	RAO 30°/CAU 30°	RAO 30°	RAO 30°/CRA 30°	LAO 10°/CRA 30°	LAO 30°/CRA 30°	LAO 45°/CRA 30°	LAO 45°	LAO 45°/CAU 30°
PA 0°	NA	–	–	–	–	–	–	–	–	–
RAO 10°/CAU 30°	3063	NA	–	–	–	–	–	–	–	–
RAO 30°/CAU 30°	1162	5381	NA	–	–	–	–	–	–	–
RAO 30°	4357	1891	3700	NA	–	–	–	–	–	–
RAO 30°/CRA 30°	1735	0	0	4778	NA	–	–	–	–	–
LAO 10°/CRA 30°	3824	0	0	943	2115	NA	–	–	–	–
LAO 30°/CRA 30°	1940	0	0	0	0	7038	NA	–	–	–
LAO 45°/CRA 30°	27	0	0	0	0	2232	7016	NA	–	–
LAO 45°	197	0	0	0	0	917	1859	2430	NA	–
LAO 45°/CAU 30°	0	0	0	0	0	0	0	0	1769	NA

CAU, caudal; CRA, cranial; LAO, left anterior oblique; PA, posterior‐anterior; RAO, right anterior oblique; *A_s_*, area of the X‐ray beam field on the actual skin surface.

### 2.4 Statistical analyses

The statistical analyses were performed using the statistical computing software R (version 3.6.1, R Foundation for Statistical Computing, Vienna, Austria).^21^ Numerical variables are reported as means and standard deviations (SDs). Comparisons of two samples were evaluated with the Student's *t*‐test, paired *t*‐test, or with the Wilcoxon rank sum test according to their characteristics. The correlation coefficients of SID, SSD, *A_p_*, and *A_s_* were determined according to the total panning, and were evaluated with the Pearson product‐moment correlation coefficient. *p* values ≤ 0.05 were considered to indicate statistically significant differences.

## 3 RESULTS

### 3.1 Total panning, SID, SSD, *A_p_*, and *A_s_*

**FIGURE 3:**
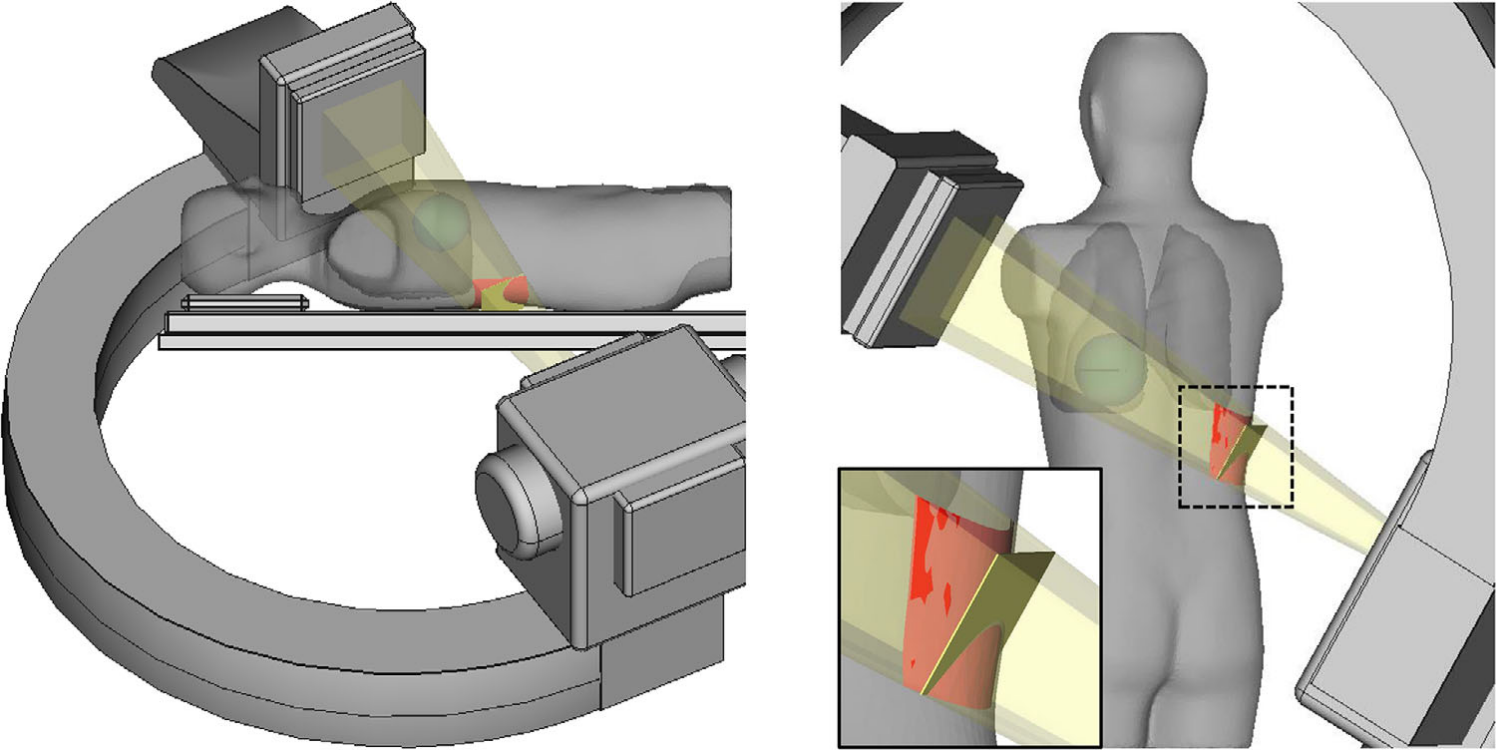
*A_p_* and *A_s_* for the male phantom in LAO 45°/CRA 30° views. The male phantom was placed on the 40 mm pad on the examination table (PERP). After the X‐ray tube angulation was set at the LAO 45°/CRA 30° view, the panning was performed so that the heart center was located in the central beam axis. After the image receptor cover and the phantom were separated by 100 mm, the values of *A_p_* (square in yellow color) and *A_s_* (rectangle in red color) were calculated and superimposed on the male phantom*. A_p_*, area of the X‐ray beam in a plane perpendicular to the beam axis at entrance skin surface; *A_s_*, area of the X‐ray beam field on the actual skin surface; CRA, cranial; LAO, left anterior oblique; PERP, patient entrance reference point

Regarding the male phantom, the total panning was increased in accordance with the steepness of the X‐ray tube angulations. Similarly, the SID was increased as the total panning increased, and the correlation coefficient was 0.849 (95% confidence interval (CI), 0.471 to 0.963, *p* < 0.01) (Table 1). Conversely, the SSD was reduced as the total panning increased (correlation coefficient = −0.861, 95% CI, −0.967 to −0.506, *p* < 0.01). The *A_p_* and *A_s_* values were both reduced as a function of the total panning, and these correlation coefficients were −0.924 (95% CI, −0.982 to −0.704, *p* < 0.001) and −0.642 (95% CI, −0.906 to −0.021, *p* < 0.05), respectively. Because the *A_s_* were obliquely calculated along the actual skin surface, the *A_s_* was 1.20 ± 0.09 times larger compared with the *A_p_* (*p* < 0.001) (Table 1 and Figure 3).

**FIGURE 4:**
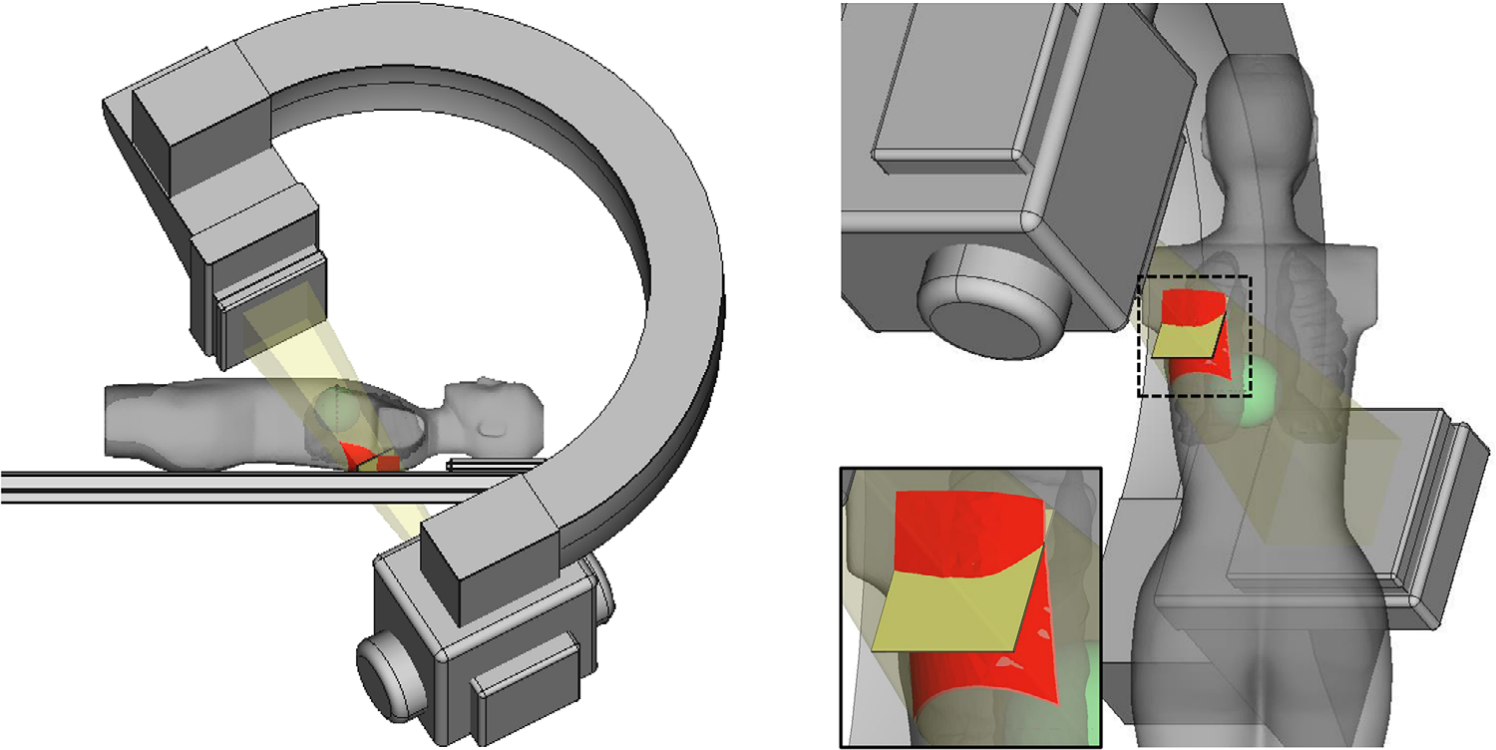
*A_p_* and *A_s_* for female phantom in RAO 30°/CAU 30° views. The female phantom was placed on the 40 mm pad on the examination table (PERP). After the X‐ray tube angulation set at the RAO 30°/CAU 30° view, the panning was performed so that the heart center was located in the central beam axis. After the image receptor cover and the phantom was separated by 100 mm, *A_p_* (square in yellow) and *A_s_* (rectangle in red) were calculated and superimposed on the female phantom. *A_p_*, area of the X‐ray beam in a plane perpendicular to the beam axis at entrance skin surface; *A_s_*, area of the X‐ray beam field on the actual skin surface; CAU, caudal; PERP, patient entrance reference point; RAO, right anterior oblique

Similarly, the SID for the female phantom was increased as the total panning increased (that was in turn increased as a function of the X‐ray tube angulation). In this case, the correlation coefficient was 0.786 (95% CI, 0.310 to 0.947, *p* < 0.01) (Table 2). Conversely, the SSD was reduced as a function of the total panning (correlation coefficient = −0.764; 95% CI, −0.941 to −0.260, *p* < 0.05). The *A_p_* was reduced with the total panning (correlation coefficient of −0.826 (95% CI, −0.958 to −0.410, *p* < 0.01). However, no statistically significant difference was found between *A_s_* and the total panning (−0.364, 95% CI, –0.808 to 0.345). Furthermore, the *A_s_* value was 1.24 ± 0.11 times larger than the *A_p_* (*p* < 0.001) (Table 2 and Figure 4).

The total panning and SID for the male phantom were larger than those for the female phantom (both *p* values < 0.05), while no statistically significant differences were found between the SSD and *A_p_* for these phantoms. The *A_s_* value for the male phantom was lesser than that for the female phantom (*p* < 0.001).

### 3.2 Overlaps of the radiation field on the skin

**FIGURE 5:**
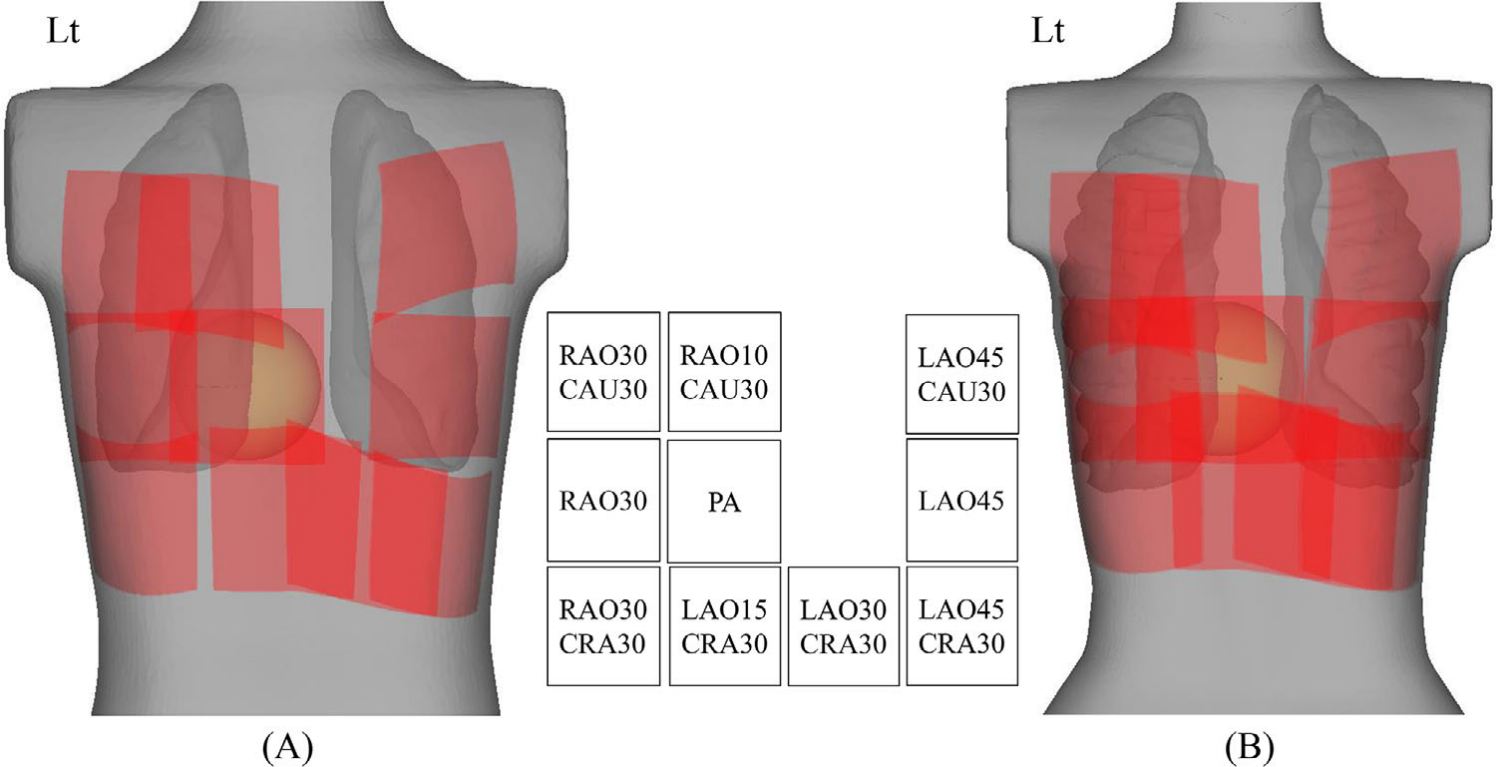
*A_s_* overlaps for male and female phantoms. *A_s_* overlaps for the male phantom (A) were smaller than those for the female phantom (B). Because these overlaps appeared on the peripheral regions of the *A_s_*, the crescent wedge filter (equalization filter) would be effective to reduce the absorbed dose to the skin at these peripheral overlap regions. *A_s_*, area of the X‐ray beam field on the actual skin surface; CAU, caudal; CRA, cranial; LAO, left anterior oblique; RAO, right anterior oblique

As mentioned in Section of Results, the *A_s_* value for the male phantom was smaller than that for the female phantom. Therefore, the *A_s_* overlaps for the female phantom were larger than those of the male phantom (*p* < 0.001) (Figure 5). The *A_s_* overlaps for the male phantom were 15/45 view combinations (33%) (Table 3), and the summation and mean ± SD of the overlaps were 23591 and 524 ± 1222 mm^2^, respectively. Similarly, the number of the *A_s_* overlaps for the female phantom were 21/45 view combinations (47%) (Table 4), and the summation and mean ± SD of the overlaps were 58374 and 1297 ± 1948 mm^2^, respectively.

## 4 DISCUSSION

Development of the format conversion from DICOM to STL data resulted in the capability of the patient‐specific modeling in the CAD software. The hollow tool could be applied for the STL data to create the cavity with the predefined wall thickness. With these technical advances, the cardiovascular angiography system and the patient with 1 mm skin could be modeled with the CAD software. CAD‐based X‐ray beam modeling exhibited several benefits that allowed the analysis of the panning, SID, SSD, *A_p_*, and *A_s_*, in conjunction with patient‐specific CT data.

Panning is a technique used to image the region‐of‐interest by moving the examination table. The heart is routinely centered in the image in PCI because the coronary arteries run along the outer surface of the heart. The panning is closely related to the heart's center‐to‐isocenter vertical distance, and increases as a function of the vertical distance. The panning for the male phantom was larger than the female phantom in this study because the vertical distance for the male phantom was larger than the female phantom (Tables 1 and 2).

The SID could be determined after the table height and panning were both defined. As predicted, the SID was increased as a function of the X‐ray tube angulation to avoid the collision with the phantom. Fukuda et al. reported that the mean and SD of SID was 1023 ± 59 mm when the same male anthropomorphic phantom was placed on the examination table of an Inova 2100 cardiovascular angiography system (GE Healthcare, Milwaukee, WI, USA).^22^ The 100‐mm separation from the image receptor cover to the phantom surface applied in this study was reasonable because there was no statistical difference between the previously reported and the currently calculated SID.

SSD may also be determined after the table height and panning were defined. As mentioned in the Introduction section, SSD could be used for the $K_{a,i}$ correction, according to Equation (1). The SPD was 550 mm in this study. The minimum and maximum SSD calculated for the male and female phantoms were 547 and 551 mm at the left anterior oblique orientation at 45°/caudal 30°, and 593 and 599 mm at posterior–anterior 0°, respectively. Therefore, the correction factor $\frac{SPD^{2}}{SSD^{2}}$ ranged from 0.84 to 1.01. This means that $K_{a,e}$ may be overestimated if the correction was not applied to the $K_{a,i}$. Furthermore, *k*_ta_ and *B* are also required for the accurate correction of $K_{a,i}$. Sadick et al. reported that the table attenuation (without the pad) varied in accordance with the X‐ray tube angulation and increased up to 25%.^23^ Alternatively, given that some manufacturers provide the *k*_ta_‐corrected $K_{a,i}$ and $P_{\mathrm{KA}}$ readout values, medical physicists should confirm the applicability of the correction factor.^24^

*A_p_* may be determined after the definitions of the table height, panning, and SID. *A_p_* initially depends on the selected FOV and the collimation at the FPD. In this study, it was set to 175 × 175 mm. Moreover, *A_p_* decreased as a function of SID because the aperture of the collimator automatically closes to fit the FOV at the FPD. Interestingly, *A_p_* in the cranial views are larger than those in the caudal views because the shape of the shoulder allows the SID in the cranial views to be smaller than the caudal views. As mentioned in the introduction section as well as SSD correction, *A_p_* could be used for the $P_{\mathrm{KA}}$ correction as shown in Equation (2). Because the ratio of the maximum to minimum *A_p_* for the male and female phantoms were relatively large and respectively equal to 1.40 and 1.43, there is a possibility of overestimating $K_{a,e}$ as well as the SSD correction. In this case, the individual *A_p_* as a function of the X‐ray tube angulation would be useful for the $P_{\mathrm{KA}}$ correction (Tables 1 and 2).

*A_s_* could be also determined after the definitions of the table height, panning, and SID. It is important to evaluate the absorbed dose to the skin and the accurate *A_p_* in PCI to avoid the radiation‐induced skin injuries. The variable $K_{a,e}$ in Equations (1) and (2) could be applied for the estimation of the absorbed dose to the skin by considering the conversion factor from $K_{a,e}$ to the absorbed dose to the skin in the clinical setting. *A_s_* also indicated where the skin injuries appeared. If the absorbed dose to skin exceeds 2 Gy and the *A_s_* is accurately determined, such as in this study, cardiologists should consider the alternation of the X‐ray angulation to avoid the *A_s_* overlap without compromising the clinical tasks. Interestingly, the *A_s_* overlaps appeared on the peripheral region of the *A_s_* (Figure 5). Therefore, the peripheral region of the absorbed dose to the skin should be reduced as much as possible. In this case, the crescent wedge filter (equalization filter) should be frequently applied to reduce the peripheral region of the *A_s_* in the clinical setting.^25^ There are two reasons for which the overlaps of the female phantom were much larger than those of the male phantom. First, there was less panning in the case of the female phantom compared with the male phantom. This is attributed to the fact that the heart center‐to‐isocenter vertical distance for the female phantom was shorter than that of the male phantom. The other reason is the fact that the *A_p_* for the female phantom was larger than the male phantom because the SID for the female phantom was smaller than that of the male phantom. As such, the patient physique would affect the amount of overlaps of the *A_s_*.

There were three limitations to this study. First, the CAD‐based X‐ray beam modeling in conjunction with the patient‐specific CT data is accurate for delineating the geometrical circumstances. However, only two male and female anthropomorphic phantoms were employed instead of the real patients in this study. Therefore, the sample size that was used to compare the physique was too small to conclude the geometrical differences according to the gender. Besides, in this modeling, it is necessary the patient‐specific CT data that are not always scanned before PCI. To overcome the issue, the phantom approximation of a patient would be an alternative feasible approach.^12^ Second, because the cardiovascular angiography system is currently not equipped with the CAD‐based X‐ray beam modeling, the geometrical analysis could only be performed in retrospective fashion. Third, the examination table and thick pad are employed in the clinical setting. Because the thick pad contributes to the alteration of the weight distribution of the patient, the supine position may lead to different results compared with CT scanning. Despite these limitations, CAD‐based X‐ray beam modeling would contribute to the analysis of the geometrical parameters in PCI.

## 5 CONCLUSIONS

A new CAD‐based X‐ray modeling technique was developed to determine the panning, SID, SSD, *A_p_*, and *A_s_* in PCI in conjunction with patient‐specific CT data. Regardless of the gender, the SID increased as a function of the total panning. However, the SSD and *A_p_* were reduced as a function of the total panning. The total panning and SID for the male phantom were larger than those for the female phantom, while the *A_s_* for the male phantom was smaller than that for the female phantom. Furthermore, the CAD‐based X‐ray beam modeling technique would help to determine the overlaps of the *A_s_*. Therefore, the cardiologists can discuss how much the X‐ray angulation needs to change to avoid the *A_s_* overlap without compromising the clinical tasks based on the CAD‐based modeling technique.

## CONFLICT OF INTEREST

The authors declare no conflict of interest.

## AUTHOR CONTRIBUTIONS

Atsushi Fukuda, PhD, Conception and design of the study, analysis and interpretation of data, collection and assembly of data, drafting of the article, and final approval of the article. Pei‐Jan P. Lin, PhD, Conception and design of the study, analysis and interpretation of data, critical revising, final approval of the article. Nao Ichikawa, MSc, Conception and design of the study, analysis and interpretation of data, collection and assembly of data, and final approval of the article. Kosuke Matsubara, PhD, Conception and design of the study, analysis and interpretation of data, and final approval of the article.

## Data Availability

Data available on request from the authors.
